# Evaluation of BMP-2 Enhances the Osteoblast Differentiation of Human Amnion Mesenchymal Stem Cells Seeded on Nano-Hydroxyapatite/Collagen/Poly(l-Lactide)

**DOI:** 10.3390/ijms19082171

**Published:** 2018-07-25

**Authors:** Shuhong Wu, Zhili Xiao, Jinlin Song, Min Li, Wenhua Li

**Affiliations:** 1 College of Stomatology, Chongqing Medical University, Chongqing 401147, China; shuhongwu069@gmail.com (S.W.); jinlinss973@gmail.com (J.S.); minlimin2006@gmail.com (M.L.); wenhuali000@gmail.com (W.L.); 2 Chongqing Key Laboratory of Oral Diseases and Sciences, Chongqing 401147, China; 3 Chongqing Municipal Key Laboratory of Oral Biomedical Engineering of Higher Education, Chongqing 401147, China

**Keywords:** human amnion mesenchymal stem cells, bone morphogenetic protein 2, nano-hydroxyapatite/collagen/poly(l-lactide) (nHAC/PLA), osteoblasts

## Abstract

Background: The aim of this study is to evaluate the effects of recombinant human bone morphogenetic protein 2 (rhBMP-2), human amnion mesenchymal stem cells (hAMSCs), and nanohydroxyapatite/collagen/poly(l-lactide) (nHAC/PLA) in tissue engineering to provide potential approaches for periodontal bone regeneration. Methods: hAMSCs were isolated from discarded amniotic membrane samples and cultured in vitro. Alkaline phosphatase (ALP) staining and alizarin red staining were performed to evaluate the osteoblast (OB) differentiation ability of hAMSCs. Three groups were divided: the experimental group (cells transfected with pcDNA3.1-rhBMP-2), the blank group (cells without gene transfection), and the control group (cells transfected with empty plasmid). RT-PCR and western blot were used to examine whether rhBMP-2 has been successfully expressed. 3-(4,5)-dimethylthiahiazol(-z-y1)-3,5-di-phenytetrazo-liumromide assay (MTT) was done to detect the effect of rhBMP-2 on hAMSCs seeded on nHAC/PLA. ALP activity, mineral formation assay, calcium, phosphate and osteocalcin (OCN) content, and OCN and RUNX2 expression of hAMSCs were detected to evaluate osteogenic differentiation capability of rhBMP-2 on hAMSCs seeded on nHAC/PLA. Results: hAMSCs exhibited intense ALP staining, obvious calcium deposition, and mineralization nodules, and rhBMP-2 were highly expressed in the experimental group. The proliferation of the hAMSCs with rhBMP-2 on nHAC/PLA was significantly higher than the cells without rhBMP-2, and the cells all increased in a time-dependent manner. rhBMP-2 significantly increased the OCN and phosphate content, mineral formation, ALP activity, osteogenic biomarkers OCN, and Runx2, and decreased calcium content in hAMSCs seeded on the nHAC/PLA scaffold. Conclusions: This finding demonstrated that hAMSCs has an ideal OB differentiation ability. rhBMP-2 facilitates the proliferation and osteogenesis of hAMSCs. The nHAC/PLA could act as a good scaffold for hAMSCs seeding, proliferation, and osteogenic differentiation. The application of rhBMP-2, nHAC/PLA, and hAMSCs in tissue engineering may offer promising possibilities for periodontal bone regeneration.

## 1. Introduction

There are two types of cells involved in bone remodeling-osteoblast (OB) and osteoclast (OC). Osteoblasts play roles in forming new bone, while osteoclasts have the function of resorbing old bone, both of which are closely connected with each other. Bone remodeling is a constant and dynamic process, which is initiated by osteoclasts. As soon as the osteoclasts attach to old bone and erode the bone matrix, the osteoblasts are recruited and begin to fill up the cavities formed by the osteoclasts, further forming new bone [1,2].

Periodontal bone defect can be caused by multiple factors, like dental caries, periodontal disease, trauma, cancer, systemic diseases, and so on [3]. Minor periodontal bone defects can repair themselves, while larger ones cannot, which may possibly lead to tooth loss or even affect mouth functioning and facial aesthetics. Currently, treatments of periodontal bone defects include autologous bone grafts, allogeneic bone grafts, xenogeneic bone grafts, and bone substitute regeneration techniques [4]. However, various problems limit the use of bone transplantation in clinics. For example, though autograft can provide osteoinductive growth factors, osteogenic cells, and an osteoconductive scaffold, the application of autologous bone grafts is still limited due to its insufficient biocompatibility, resorption of bone, residual pain, limited graft quantity, and donor-site morbidit [5,6,7]. Besides, the trauma caused by autograft is great, the operation risk is relatively high, and the operation time is rather long [8,9]. Allogeneic or xenogeneic bone grafts may result in rejection, disease transmission, and bone nonunion. Before allograft, all immunogenic proteins are supposed to be removed to avoid any risk of immunogenic reaction by techniques such as lyophilization, irradiation, or freeze-drying [10,11]. In addition, all kinds of bone substitute are expensive, and their regenerative ability is limited.

Because of these problems of natural bone grafting, tissue engineering recently has been drawing more and more attention as a promising alternative to natural bone grafts. Tissue engineering induces bone tissue regeneration mainly via a composite graft containing osteoinductive growth factors and osteogenic cells along with a synthetic osteoconductive matrix substitute. A suitable scaffold can provide an appropriate environment for osteogenic cells to migrate, proliferate, differentiate, and promote new bone formation. Therefore, an ideal scaffold and an osteogenic cell are two pivotal factors for the successful engineering of alveolar bone tissues.

Recently, a developed ceramic/polymer composite material, nano-hydroxyapatite/collagen/poly(l-lactide) (nHAC/PLA), with properties similar to natural bone, has drawn increasing attention. It is considered a bone substitute. Natural bone is composed of hydroxyapatite [Ca_10_(PO_4_)_6_(OH)_2_] crystals with low crystallinity and nanometer size deposited within an organic matrix (~95% is type I collagen) [12]. nHAC, prepared by mineralizing type I collagen, exhibits similar features to natural bone in terms of both composition and hierarchical structure [13]. Previous studies have demonstrated that the composite material is highly osteoconductive, biocompatible, and bioresorbable in both cell culture and animal model experiments [14,15]. Ling reported that nHAC/PLA has been employed in periodontal tissue engineering. However, whether nHAC/PLA can be used in human mesenchymal stem cells is still unclear [16].

There are some cell types with potential tissue regeneration capability, such as embryonic stem cells, hematopoietic stem cells, and mesenchymal stem cells (MSCs). Among them, MSCs from bone marrow would be an ideal candidate for tissue engineering, because they have rich differentiation potential and anti-inflammatory capacity. However, they have the disadvantage of relatively low abundance and pain association with their extraction. Human amnion mesenchymal stem cells (hAMSCs) obtained from human amniotic membrane are easily available and abundant. They overcame the defects of MSCs and possess easy extraction and high abundance. Meanwhile, they have similar phenotypes and an equivalent differentiation potential to their homologues from the bone marrow [17,18,19]. They also have the characteristic of not being likely to have oncogenic transformation after cultivation and implantation in vitro [20]. However, to our knowledge, the behavior of hAMSCs on nHAC/PLA scaffold have not yet been fully elucidated.

There are several kinds of osteoinductive growth factors to induce bone regeneration in the body, including bone morphogenetic protein (BMP), transforming growth factor β, and basic fibroblast growth factor [21]. Bone morphogenetic protein 2 (BMP-2) is one of the osteoinductive growth factors, which mainly influences bone and hematopoietic cell differentiation [22,23]. Because of the characteristics of inducing migration and differentiation of MSCs toward the osteogenic phenotype [24,25], BMP-2 has been applied in bone repair and regeneration therapy [26]. Chen et al. [16] once studied the capacity of the combination of nHAC/PLA, dental pulp stem cells, and BMP-2 on the reconstruction of critical-size alveolar bone defects. Nonetheless, the study of BMP-2 and nHAC/PLA on osteogenesis of hAMSCs are still limited.

In the present study, we cultured hAMSCs transfected with recombinant human BMP-2 (rhBMP-2) on nHAC/PLA scaffold. The cell proliferation, osteogenic capacity and biocompatibility of the constructs were evaluated in vitro. Our data shown that nHAC/PLA material scaffold promoted bone formation in the periodontal defect, which can obtain partial periodontal tissue regeneration, and early repair of tooth extraction trauma, these promote early healing of periodontal ligament and alveolar bone, may possibly provide a potential approach for functional periodontal bone regeneration.

## 2. Results

### 2.1. Cultural and OB Differentiation Identification of hAMSCs

**Figure 1:**
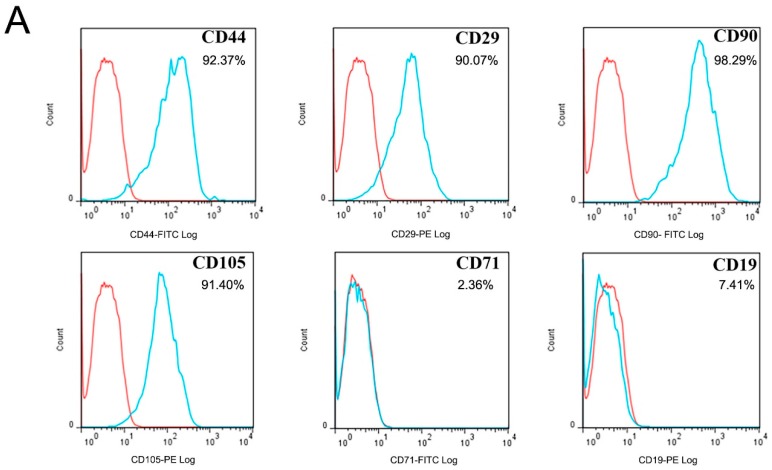

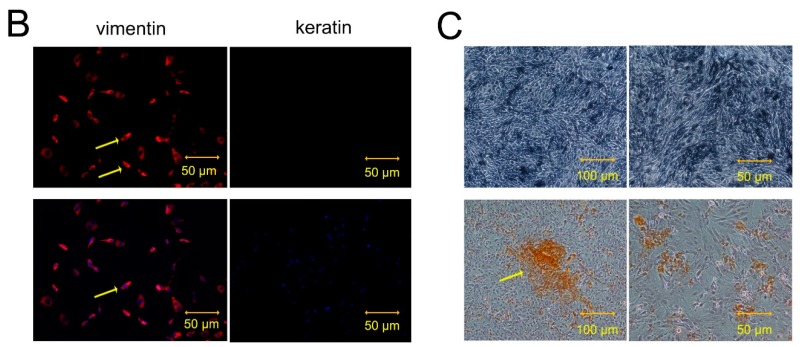
Identification and immunohistochemical staining figures of hAMSCs cells. (**A**) Flow cytometry was used to detect the expression of CD markers. The results showed that hAMSCs cells expressed CD44, CD29, CD90, and CD105 but not CD71, CD19. The red line is the same type of control, and the blue line is the actual expression. (**B**) Immunofluorescence staining display hAMSCs cells expressed vimentin (red) and unexpressed keratin (nucleus staining blue, DAPI). (**C**) An intense staining for ALP was observed after being cultured in osteogenic medium for 2 days. The staining for ALP became much more intense after being cultured in osteogenic medium for 5 days. Obvious calcium deposition (red) and mineralizing nodules (arrows) are visible by alizarin red staining.

Flow cytometry was used to detect the expression of CD markers. The results showed that hAMSCs cells expressed CD44, CD29, CD90, and CD105, but not CD71 or CD19 (Figure 1A). Immunofluorescence staining revealed that hAMSCs cells express vimentin (red) but do not express keratin (Figure 1B). Then, we performed ALP staining and alizarin red staining to measure hAMSCs osteogenic capability. After being cultured in osteogenic medium, there was intense staining for ALP, and alizarin red staining results exhibited obvious calcium deposition and mineralization nodules (Figure 1C). These observations demonstrate the OB differentiation ability of hAMSCs, which could possibly be used for tissue regeneration.

### 2.2. Identification of the Recombinant Plasmid

**Figure 2:**
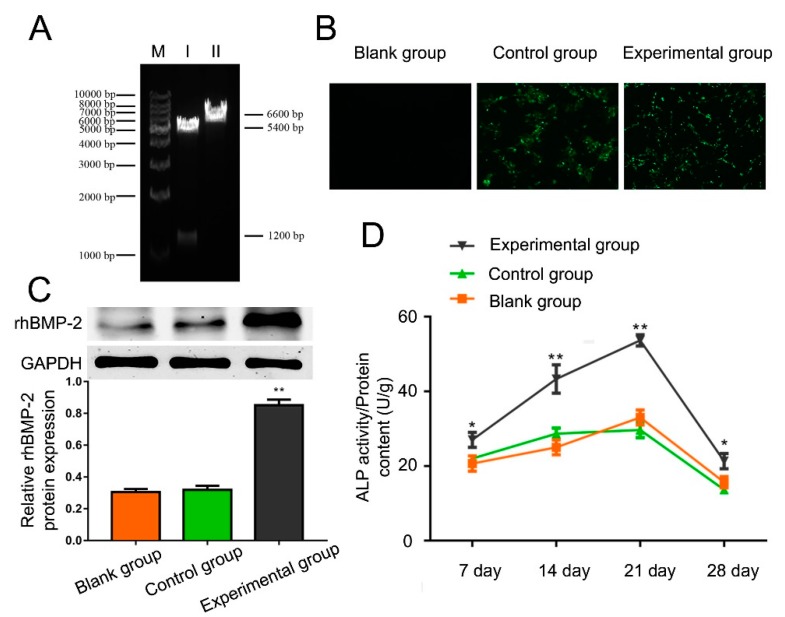
(**A**) Recombinant plasmid pcDNA3.1-rhBMP-2 electrophoresis. M: DNA ladder marker with relative molecular weight 1 kbp; I: the recombinant plasmid exhibited two bands at 1.2 and 5.4 kbp with dual-enzyme digestion; II: the recombinant plasmid exhibited a band at 6.6 kbp (a recombinant plasmid pcDNA3.1-rhBMP-2) without dual-enzyme digestion. (**B**) Analysis of green fluorescent protein (GFP) expression showed equal transfection efficiency for both control (empty vector) and experiment group (rhBMP-2 containing vector). (**C**) Western blot results demonstrated that the expression of rhBMP-2 was remarkedly increased in the experimental group compared with the blank and control groups. (**D**) hAMSCs transfected with rhBMP-2 significantly increased the ALP activity compared with the blank group and the control groups at each time point. ($\bar{x}$ ± s, *n* = 3, * *p* < 0.05, ** *p* < 0.01).

The recombinant plasmid with dual-enzyme digestion exhibited two bands at 1.2 and 5.4 kbp (1.2 kbp was rhBMP-2-cDNA product and 5.4 kbp was pcDNA3.1 linear plasmid) separately (Figure 2A). Without dual-enzyme digestion, the recombinant plasmid exhibited a band at 6.6 kbp (a recombinant plasmid pcDNA3.1-rhBMP-2), indicating that the recombinant plasmid pcDNA3.1-rhBMP-2 was successfully constructed. 

### 2.3. Cell Transfection Efficiency

48 h after transfection, green fluorescence was observed in both the experimental group and the control groups, but there was no fluorescence observed in the blank group (Figure 2B). Western blot results demonstrated that the expression of rhBMP-2 increased remarkably in the experimental group compared with the blank and control groups (Figure 2C), revealing that rhBMP-2 expressed in the experimental group was significantly higher than the blank and control groups. These results suggest that the rhBMP-2 genes were successfully expressed.

### 2.4. Effect of rhBMP-2 on ALP Activity of hAMSCs

The ALP activity was analyzed in the experimental group, the blank group, and the control groups after 7, 14, 21, and 28 days (Figure 2D). The ALP activity of each group reached the highest level at day 21. hAMSCs transfected with rhBMP-2 significantly increased the ALP activity compared with the blank group and the control groups at each time point. These data demonstrate the osteogenic effect of rhBMP-2 on hAMSCs.

### 2.5. Scanning Electron Microscope (SEM) Analysis

**Figure 3:**
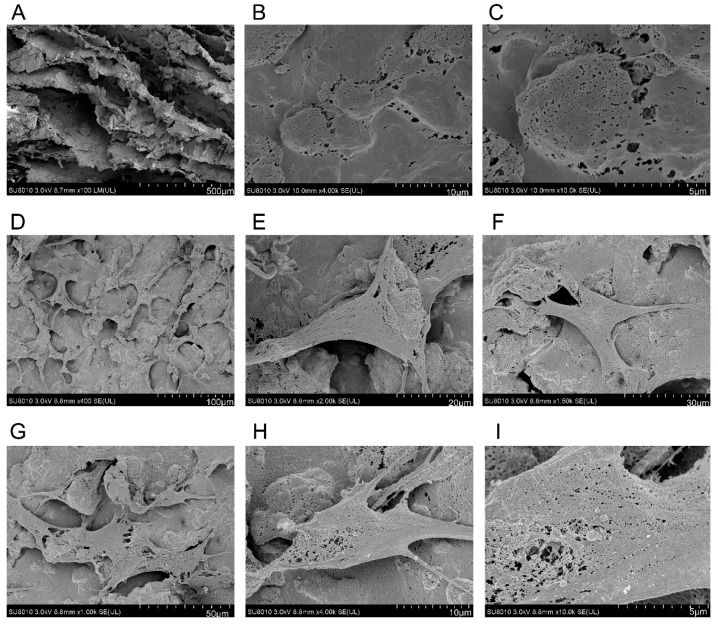
(**A**) Scanning electron microscope showing that the nHAC/PLA blocks were similar to the natural bone in terms of microstructure of natural bone in both main component and hierarchical microstructure. (**B**,**C**) the hAMSCs adhered to, extended, and produced a few extracellular matrix (ECM) on the nHAC/PLA surface after five days of cultivation. (**D**–**H**) The hAMSCs were in triangle, cube shape, or spindle shape with developed cytoplasmic extensions attached to the scaffold. (**I**) hAMSCs dramatically proliferated in the pore of the scaffold material and linked flakiness on the surface after 28 days cultivation. The cells became confluent, forming a distinct multilayer, and some cells were covered with deposits.

The adherence and morphology of hAMSCs seeded on nHAC/PLA were observed by SEM (Figure 3A–I). The nHAC/PLA blocks were similar to the natural bone in terms of microstructure of natural bone in both main component and hierarchical microstructure. After five days of culture, the hAMSCs adhered to, extended, and connected with each other and produced a few extracellular matrix (ECM) on the nHAC/PLA surface. The hAMSCs were in spindle, triangle, or cube shape, short fusiform-shape with developed cytoplasmic extensions attached to the scaffold. After 28 days cultivation, they had dramatically proliferated in the pore of the scaffold material and attached to link flakiness on the surface. The cells became confluent, forming a distinct multilayer, and some cells were covered with deposits. All these observations proved good biocompatibility between hAMSCs and nHAC/PLA.

### 2.6. Effect of rhBMP-2 on Proliferation in the hAMSCs Seeded on the nHAC/PLA

**Figure 4:**
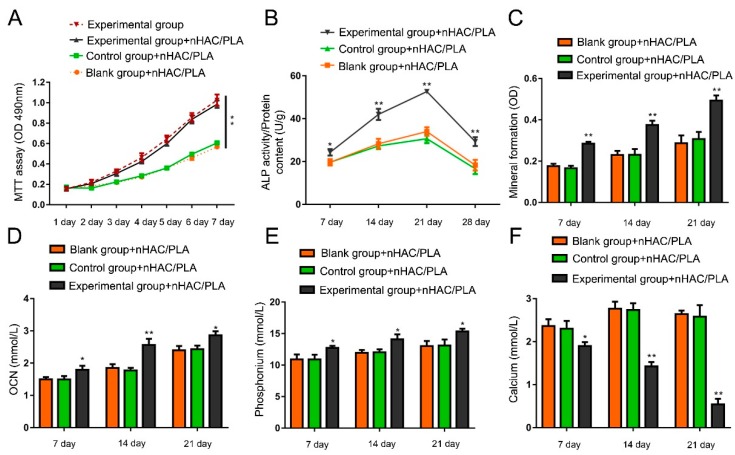
(**A**) Effect of rhBMP-2 on proliferation in the hAMSCs seeded on the nHAC/PLA. The proliferation of the hAMSCs with rhBMP-2 is significantly higher than the cells without rhBMP-2. (**B**) Effect of rhBMP-2 on ALP activity of hAMSCs seeded on nHAC/PLA. (**C**) Effect of rhBMP-2 on mineral formation in the hAMSCs seeded on the nHAC/PLA. (**D**–**F**) Effect of rhBMP-2 on OCN, phosphate, and calcium content of hAMSCs seeded on nHAC/PLA. (**D**,**E**) The OCN and phosphate content in the experimental group of hAMSCs on nHAC/PLA were remarkedly higher compared with the control and blank control groups of hAMSCs seeded on nHAC/PLA during the 21 days of culture. (**F**) The calcium content in the experimental group of hAMSCs on nHAC/PLA was significantly lower than the control and blank control groups of hAMSCs on nHAC/PLA during the 21 days of culture. ($\bar{x}$ ± s, *n* = 3, * *p* < 0.05, ** *p* < 0.01).

MTT assay was used to detect the proliferation of hAMSCs seeded on the nHAC/PLA. The results showed that the proliferation of the hAMSCs with rhBMP-2 is significantly higher than the cells without rhBMP-2 (Figure 4A). In these three groups seeded on nHAC/PLA scaffolds, the proliferation of hAMSCs all increased in a time-dependent manner, indicating that nHAC/PLA has no negative effect on proliferation. Though compared with cells without nHAC/PLA scaffolds, the number of hAMSCs on nHAC/PLA with or without rhBMP-2 both decreased, but there was no significant difference. These data imply that nHAC/PLA could probably be used as an ideal tissue engineering scaffolds.

### 2.7. Effect of rhBMP-2 on ALP Activity of hAMSCs Seeded on nHAC/PLA

The ALP activity was analyzed among the three groups of hAMSCs seeded on nHAC/PLA after 7, 14, 21, and 28 days (Figure 4B). The ALP activity of each group reached the highest level at day 21. The experimental group of hAMSCs with high expression of rhBMP-2 seeded on nHAC/PLA significantly increased the ALP activity compared with the control and blank groups of hAMSCs seeded on nHAC/PLA at each time point. These results suggest that rhBMP-2 is involved in hAMSCs osteogenesis.

### 2.8. Effect of rhBMP-2 on Mineral Formation in the hAMSCs Seeded on the nHAC/PLA

Alizarin red staining was used to quantify calcium phosphate mineral formation of the three groups of hAMSCs on nHAC/PLA following culture for 21 days (Figure 4C). Compared with the control and blank groups of hAMSCs seeded nHAC/PLA scaffolds, the mineral formation of the experimental group of hAMSCs on nHAC/PLA was significantly higher, indicating that rhBMP-2 plays an important role in hAMSCs osteogenesis.

### 2.9. Effect of rhBMP-2 on Calcium, Phosphate, and OCN Content of hAMSCs Seeded on nHAC/PLA

OCN, phosphate, and calcium content were measured to investigate the osteogenic capability of hAMSCs seeded on nHAC/PLA with or without high rhBMP-2 expression after 7, 14, and 21 days (Figure 4D–F). During the 21 days of culture, the OCN and phosphate content in both groups gradually increased in a time-dependent manner and the OCN, and phosphate contents in hAMSCs seeded on nHAC/PLA with high rhBMP-2 expression were remarkedly higher compared with the control and blank control groups of hAMSCs seeded on nHAC/PLA. On the contrary, the calcium content in both groups declined, and the content in the experimental group of hAMSCs on nHAC/PLA was always significantly lower. These results show that rhBMP-2 enhanced osteogenic capability of hAMSCs seeded on nHAC/PLA.

### 2.10. Effect of rhBMP-2 on OCN, RUNX2, ALP, and COL1A1 Expression of hAMSCs Seeded on nHAC/PLA

**Figure 5:**
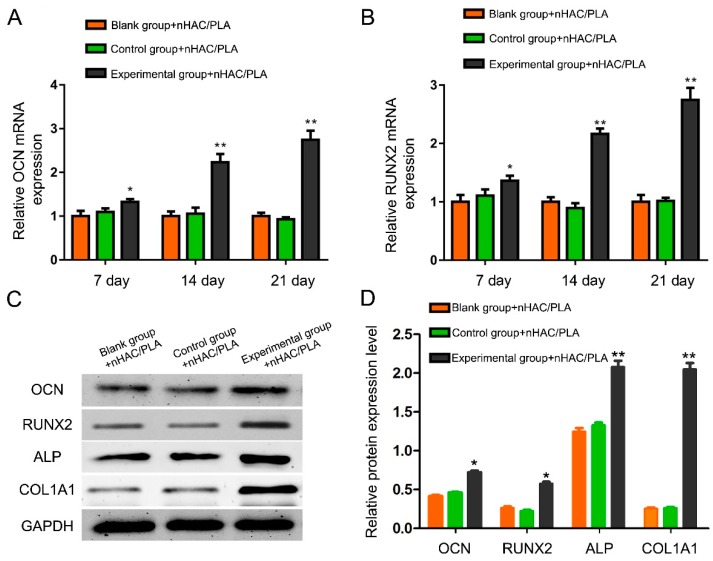
Effect of rhBMP-2 on OCN and RUNX2 expression of hAMSCs seeded on nHAC/PLA. (**A**,**B**) The mRNA expression of OCN and RUNX2 from the experimental group of hAMSCs seeded on nHAC/PLA was markedly up-regulated compared with cells in the control and blank control groups by Real-time PCR. (**C**,**D**) Western blot results demonstrated that the expression of OCN, RUNX2, ALP, and COL1A1 proteins from the experimental group of hAMSCs on nHAC/PLA were significantly increased compared with cells in the control and blank control groups. Western blot results were normalised to glyceraldehyde phosphate dehydrogenase (GAPDH) expression. ($\bar{x}$ ± s, *n* = 3, * *p* < 0.05, ** *p* < 0.01).

RT-qPCR was used to reveal the mRNA expression levels of OCN and RUNX2 in the hAMSCs seeded on the nHAC/PLA among three groups. The mRNA expression of OCN and RUNX2 from the experimental group of hAMSCs seeded on nHAC/PLA was markedly up-regulated compared with cells in the control and blank control groups (Figure 5A,B). Western blot was used to analyze the protein expression of these OB function markers, and the results were consistent with the mRNA expression, suggesting that rhBMP-2 participates in osteogenesis of hAMSCs on nHAC/PLA (Figure 5C,D).

## 3. Discussion

Autologous adult stem cell-based tissue engineering provides a promising substitute for resolving the difficulties that autologous grafting has faced for periodontal bone repair. However, the investigations on tissue engineering to reconstruct periodontal bone defect remain rare. In present study, we demonstrated that hAMSCs have ideal OB differentiation ability. rhBMP-2-transfected hAMSCs seeded on nHAC/PLA has good biocompatibility and osteogenic abilities, which may facilitate the reconstruction of periodontal bone defect.

Successful tissue regeneration demands a sufficient cell population with high differentiation potential. Among the seed cells, hAMSCs holds great potential for promoting bone formation. The sources of hAMSCs are abundant, and they can be induced to multiple mesenchymal tissues. Besides, there are rare ethical issues on them with low immunogenicity and anti-inflammatory properties [27]. Additionally, hAMSCs have the capacity to differentiate between various lineages including adipogenic, chondrogenic, and osteogenic lineages in response to external cues. In our study, we proved the OB differentiation ability of hAMSCs, which had intense ALP staining and obvious calcium deposition and mineralization nodules after being cultured in osteogenic medium for 28 days.

BMP is one of the family members of transforming growth factor-β (TGF-β), playing an important role in bone formation [28]. BMP-2, belonging to the BMPs family, is famous as a growth factor in bone regeneration because of its particularly strong osteoinductive function [29,30]. Accumulating studies have proved that BMP-2 is involved in bone formation, bone remodeling, bone development, and osteoblast differentiation [31]. Samara et al. [32] revealed that BMP-2 could induce ectopic cartilage and bone formation by implanting into muscles, and the BMP signals control the differentiation of osteoblasts, proliferation, and differentiation of chondrocytes and bone quality. Hwang et al. [33] report that new bone formation is accelerated after the usage of rhBMP-2 in patients after maxillofacial cyst enucleation. Li et al. [34] reported that rhBMP-2 enhances bone formation through the application of a carrier like fibrin during distraction osteogenesis in rabbits. Chatzinikolaidou et al. [35] suggested that bioactive rhBMP-2 release from the hybrid scaffolds can enhance osteogenesis during cell culture. BMPs have been reported to successfully heal critical-sized mandible and calvarial defects [36], and, further, the use of rhBMP-2 has been approved by FDA for regenerative therapy of spinal fusion, alveolar ridge augmentation, and sinus floor augmentation [37]. After that, rhBMP-2 began to be transfected into cells to further explore its functions in osteogenesis. Tsuda et al. [38] demonstrated that MSCs transfected with the rhBMP-2 gene can increase osteogenic activity and increase the quantity of new bone formation. Jang et al. [39] reported that rhBMP-2 enhances the osteogenesis of demineralized bone matrix in the mastoid obliteration model. In present study, we transfected rhBMP-2 into hAMSCs to investigate the role of BMP-2 on hAMSCs osteogenesis. The expression of rhBMP-2 in the experimental group was significantly higher than the blank and control groups, indicating that rhBMP-2 was successfully expressed in the experimental group. The following results showed that BMP-2 significantly enhanced the osteogenic capability of hAMSCs by elevating OCN and phosphate content, lowering calcium content, promoting mineral formation, increasing ALP activity, and up-regulating the expression of OB function markers.

The nHAC/PLA scaffold has good biocompatibility and osteoconductive capacity [40]. It was formed from new mineralized collagen consisting of a combination of collagen fibrils and PLA attached to nanohydroxyapatite by a self-assembly method. The microstructure of nHAC/PLA was similar to the hierarchical structure of natural bone [41]. In this study, SEM results exhibit that the hAMSCs adhered to, extended, and connected with each other, retaining their osteogenic phenotype on nHAC/PLA. Then, the proliferation and osteogenic capabilities of the hAMSCs on the nHAC/PLA scaffold were further evaluated. The number of hAMSCs seeded on the nHAC/PLA increased in a time-dependent manner and has no significant difference compared with cells cultured in osteogenic medium without nHAC/PLA, indicating that there was no negative effect of nHAC/PLA on proliferation. The boost of the OCN and phosphate content, the promotion of mineral formation, the increase of the ALP activity, the elevation of OCN and RUNX2 expression, and the decrease of calcium content all proved the fact that the osteogenic potential of hAMCs transfected with rhBMP-2 on nHAC/PLA was activated by the osteogenic medium.

Meanwhile, ALP activity and mineral formation ability were used to measure OB differentiation and mineralization, which are important parameters in evaluating osteogenic differentiation [42]. Guler et al. [43] observed enhanced calcium mineralization and higher ALP activity in cells after a 14-day in vitro culture of nanofibres with immobilized BMP-2, suggesting that BMP-2 biofunctionalized nanofibres could enhance in vitro osteogenic activity. Lin et al. [44] revealed that there was increased calcium accumulation, higher ALP activity, and more bone formation in a demineralized bone matrix sequentially treated with heparin and rhBMP-2. In our study, there was enhanced mineralization ability and higher ALP activity observed in the experimental group seeded on nHAC/PLA than the blank and control groups seeded on nHAC/PLA.

OCN and RUNX2 are typical OB function markers, which play important roles in osteogenesis [45,46]. As an early-stage osteoblastic differentiation marker, overexpression of RUNX2 is able to enhance hAMSCs OB differentiation to increase new bone formation. Besides, RUNX2 is a major target of the BMP pathway and is degraded by mediation of an ubiquitination pathway [47]. Different from RUNX2, OCN is a late-stage osteoblastic differentiation marker. In current study, we observed an elevated expression of OCN and RUNX2 in hAMSCs transfected with rhBMP-2 on nHAC/PLA compared with the blank and control groups seeded on nHAC/PLA.

Though in vitro studies have been comprehensively performed to elucidate the role of rhBMP-2 in the osteogenesis of hAMSCs on nHAC/PLA, another animal bone defect model was needed to characterize the osteogenic role of hAMSCs transfected with BMP-2 seeded on nHAC/PLA in vivo, further proving the feasibility of its application in periodontal tissue engineering.

In conclusion, hAMSCs has ideal OB differentiation ability, and rhBMP-2 facilitates the proliferation and osteogenesis of hAMSCs. nHAC/PLA could act as a good scaffold for hAMSCs seeding, proliferation, and osteogenic differentiation. The application of rhBMP-2, nHAC/PLA, and hAMSCs in tissue engineering may offer promising possibilities for periodontal bone regeneration.

## 4. Materials and Methods

### 4.1. Cell Isolation and Cultures

We isolated hAMSCs from discarded amniotic membrane samples and collected cells using the pancreatin/collagenase digestion method, as described previously [48]. Briefly, the amnion was manually separated from the chorion, washed extensively in phosphate-buffered saline (PBS) containing 100 U/mL penicillin and 100 g/mL streptomycin, and cut into small pieces (1 × 1 mm^2^). The minced amnion was digested with 0.25% trypsin (Gibco, Carlsbad, CA, USA) and collagenase I (0.75 mg/mL) in Dulbecco’s Modified Eagle’s Medium (DMEM; Gibco) for 60 min at 37 °C. The harvested cells were cultured in DMEM complete medium composed of DMEM medium supplemented with 10% heat-inactivated fetal bovine serum (FBS), 100 U/mL penicillin, 100 g/mL streptomycin, and 2 mM l-glutamine (Gibco) at 37 °C in a humidified atmosphere with 5% CO_2_. The passages 3rd to 7th cells were used in this study.

### 4.2. Flow Cytometry and Immunofluorescence

1 × 10^6^ 6th generation hAMSCs were harvested and digested with trypsin and washed three times with PBS. Primary antibody were added after 30 min. After washing with PBS, IgGl-FITC and IgGl-PE were incubated for 30 min. The cells stained with specific antibodies to CD44-FITC (Catalog#FC00052-FITC, P16070), CD29-PE (Catalog#V00772), CD90-FITC (Catalog#FC01818-PE), CD105-PE (Catalog#PA1395), CD71-FITC (Catalog#M00591-1), and CD19-PE (Catalog#A00154-2). All the flow cytometry antibodies were purchased from Santa Cruz Biotechnology (Dallas, TX, USA), and analyzed using Cell Quest software (BD, New York, NY, USA).

The hAMSCs in logarithmic growth phase of P3/P7 generation were prepared as single cell suspensions. The cell density was adjusted to 1 × 10^5^ cells/mL, and 1 mL per well was inoculated into a 6-well cell culture plate (with a built-in 18 mm diameter circle). After 7 days, the cells were evenly covered on the coverslips, rinsed three times in PBS, then fixed in 4% paraformaldehyde (containing 0.1 mol/L PBS) for 10 min. 0.5% Triton X-100 for 30 min, drops of Keratin antibody (1:200) and vimentin antibody (1:200), primary antibody overnight, using the corresponding secondary antibodies, mounting slides, and laser confocal microscopy to observe cell staining.

### 4.3. Construction of pcDNA3.1-rhBMP-2

According to the cDNA nucleotide sequence (NM001200), the primer of rhBMP-2 (supplied by GenBank) with added BamHI and EcoRI restriction sites was designed. The primer was as follows: forward 5′-TGGATCCTGACTCACGTCGGTCCTGT-3′, reverse 5′-GCGACACCCACAACCCTCC-3′. cDNA obtained from osteosarcoma tissue was used as a template for PCR amplification. After that, the rhBMP-2 target gene fragment was connected with the plasmid vector through ligases and cloned into the pcDNA3.1vector. The resulting product contained the plasmid of pcDNA3.1-rhBMP-2, which was determined by dual-enzyme digestion and sequencing.

### 4.4. Transfection Efficiency Detection

hAMSCs were transfected with the expression vector (pcDNA3.1-rhBMP-2) containing the rhBMP-2 gene according to the Lipofectamine2000TM transfection kit (Gibco) specifications. hAMSCs were randomly divided into three groups, an experimental group (cells transfected with pcDNA3.1-rhBMP-2), a blank group (cells without gene transfection), and a control group (cells transfected with empty plasmid). hAMSCs were cultured in 6-well culture plates, and the culture medium was changed with an Opti-MEMI optimized cell culture medium after the cells were spread over a single layer. 4 days later, a mixture of liposome and the rhBMP-2 gene was transfected in the experimental group. Then, we added the Opti-MEMI optimized cell culture medium and liposome which was diluted with Opti-MEMI optimized cell culture medium, and the volume was the same as the experimental group to the control and blank group separately. Then, the cells were incubated in a 5% CO_2_ environment at 37 °C for 4–6 h. Sequentially, the culture medium was changed to a normal complete culture medium. 24 h after transfection, the previous medium was changed with a fresh culture medium. 48 h after transfection, a microscope (Olympus, Tokyo, Japan) was used to observe the expression of green fluorescent protein (GFP) in these three groups.

### 4.5. Biometrics Preparation and Seeding of nHAC/PLA Scaffolds

The nHAC/PLA material had similar features to the natural bone composition: the porosity was 70–90%, and the pore size was (300–400) ± 150 μm [49]. The nHAC/PLA material was purchased from Beijing Allgens Medical Science & Technology Co., Ltd., Beijing, China. The hAMSCs from the experimental group, blank group, and control group were seeded onto nHAC/PLA scaffolds (Beijing Allgens Medical Science & Technology Co., Ltd.), which had been cut into 10 × 5 × 4 mm^3^ blocks. The constructs were firstly incubated in basic media in 24-well plate for 2 h at 37 °C. After the cells had adhered to nHAC/PLA, the culture medium was changed to osteogenic induction medium for in vitro characterization.

### 4.6. Scanning Electron Microscopy (SEM)

After cultivation of hAMSCs on nHAC/PLA scaffolds in osteogenic induction medium for 7 days, the samples were washed with phosphate buffered saline (PBS) and fixed in 2.5% glutaraldehyde. Then, the samples were coated with several nanometer-thick layers of gold, and a variable-pressure scanning electron microscope (S-3400N, Tokyo, Japan) with beam energies of 6–25 kV was employed to observe the adhesion and morphology of the hAMSCs on the surface of the nHAC/PLA composite.

### 4.7. Alkaline Phosphatase Staining and Activity Assay

Alkaline phosphatase (ALP) staining was performed following the protocol of the NBT/BCIP staining kit (Aibosi, Shanghai, China). The cultured cells were rinsed with PBS and fixed in 4% paraformaldehyde for 30 min. Then, the cell layer was washed three times with PBS and incubated in alkaline solution for 20 min at 37 °C. An ALP activity colorimetric assay kit (Biovision, Milpitas, CA, USA) was used to analyze ALP activity. The cultured cells were rinsed three times with PBS, followed by 1% Triton X-100, and then scraped into distilled water. After that, three cycles of freezing and thawing were done. Bicinchoninic acid (BCA) method was used to determine the total protein content in the same sample using the Pierce protein assay kit (Aibosi, Shanghai, China). ALP activity relative to the amount of total protein in the sample was calculated.

### 4.8. Alizarin Red Staining and Quantification

After the cultured cells were taken out of the incubator, the culture media were discarded and changed to mineralized culture media (α-MEN culture medium containing 8% FBS, 10^−7^ mol/L dexamethasone, 50 μg/mL ascorbic acid Vc and 10 mmol/L β-sodium glycerophosphate). The cells were cultured for another 21 days and stained with alizarin red when cells were stratified and black nodules were observed. Following the discard of the supernatant, the remaining solution was washed twice with PBS and then dried. Filtered 4% paraformaldehyde was added to fix the solution for 8 min, and then the paraformaldehyde was removed. Then, the remaining solution was washed with PBS and dried. Alizarin red was added, and the mixture was cultured for 18 min in a CO_2_ incubator. After incubation, the mixture was washed with PBS and photographed by a microscope (Olympus, Japan). Image-Pro Plus5.0 (Media Cybernetics, Rockville, MD, USA) was used to calculate the area of the mineralized nodules.

### 4.9. MTT Assay

3-(4,5)-dimethylthiahiazol(-z-y1)-3,5-di-phenytetrazo-liumromide (MTT; Sigma, Santa Clara, CA, USA) assay was used to evaluate cell proliferation ability. The experimental group, blank group, and control group of hAMSCs seeded on nHAC/PLA scaffolds were cultured in 96-well culture plates. The hAMSCs transfected with rhBMP-2 cultured in the osteogenic medium without nHAC/PLA was another control group. 20 μL MTT solution (5 g/L) was added to each tested well from 0 day to 7 days after cultivation. Subsequently, cultivation was continued for another 4 h at 37 °C. Then, discard the culture solution and add 20 μL DMSO to each tested well. A microtiter plate reader (Thermo Electron Corporation, Helsinki, Finland) was used to measure the optical density (OD) of each tested well.

### 4.10. Quantitative Real-Time Polymerase Chain Reaction

TRIzol (Invitrogen, Grand Island, NY, USA) was used to extract total RNA, and a cDNA synthesis kit (Promega, Madison, WI, USA) was used to synthesize the first-strand complementary DNA (cDNA). Quantitative real-time PCR (qRT-PCR) was performed using human osteocalcin (OCN), Runx2, ALP, BMP-2, and GAPDH primers and Fast SYBR Green MasterMix in a StepOnePlus^TM^ Real-Time PCR System (Applied Biosystems, Carlsbad, CA, USA). Each sample was done in triplicate. The mean cycle threshold (Ct) value of each target gene was normalized against Ct value of GAPDH and the relative expression calculated using the following formula: 2^−(normalized average Cts)^ × 10^4^. The oligonucleotide sequences were as follows: BMP-2, forward 5′-GGGCATCCTCTCCACAAA-3′, reverse 5′-GTCATTCCACCCCACGTC-3′; ALP, forward 5′-TCC CAC GTT TTC ACG TTT-3′, reverse 5′-GAG ACG TTC TCC CGT TCA C-3′; OCN, forward 5′-GGAGGGCAGCGAGGTAGTGAAG-3′, reverse 5′-GATGTGGTCA GCCAACTCGTCA-3′; Runx2, forward 5′-CGGAATGCCTCTGCTGTTATGAA-3′, reverse 5′-AGGATTTGTGAAGACGGTTATGG-3′; GAPDH, forward 5′-GAAGGTGAAGGTCGGAGTC-3′, reverse 5′-GAAGATGGTGATGGGATTTC-3′.

### 4.11. Western Blot Analysis

We extracted proteins from hAMSCs and digested the cells with cell lysis buffer (0.1 mol/L NaCl, 0.01 mol/L Tris/HCl (pH 7.6), 0.001 mol/L Ethylene Diamine Tetraacetic Acid (EDTA) (pH 8.0), 1 μg/L aprotinin and 100 μg/L phenylmethylsulphonyl fluoride) for 30 min, and the supernatant was obtained after centrifugation at 1200 rpm for 5 min. Coomassie Brilliant Blue method was used to detect the amount of proteins and an equal volume of sampling buffer was added. Then, the resultant was boiled for 5 min and then sampled at 100 μg per strip. Subsequently, sodium dodecyl sulphate polyacrylamide gel electrophoresis (SDS/PAGE) and western blotting assay were performed. The membrane was blocked with 5% nonfat milk in PBST for 1 h at room temperature. Then, membrane was probed with rabbit anti-human monoclonal antibody rhBMP-2 (1:1000, Aibosi, Shanghai, China), OCN (1:1000, Abcam, Shanghai, China), RUNX2 (1:1000, Cell Signaling Technology, Beverly, MA, USA), or GAPDH (1:10,000, Santa Cruz Biotechnology, Dallas, TX, USA) and was incubated overnight at 4 °C. Then, the membrane was incubated with secondary antibody conjugated with horseradish peroxidase (1:10,000; Santa Cruz Biotechnology) for 1 h at room temperature after extensive washing. Protein bands was visualized by Odyssey Infrared Imaging System (LI COR, Inc., Lincoln, NE, USA). Image J software version 1.47 (Bethesda, MD, USA) was used to quantify the relative intensity of protein bands compared with GAPDH.

### 4.12. Calcium, Phosphate, and OCN Content Assays

hAMSCs seeded on nHAC/PLA scaffolds were cultured, and the supernatants were collected on days 7, 14, and 21. Roche Diagnostics Ca/P kits was used to detect calciumcontent and phosphate content, while N-MID osteocalcin kits was used to detect OCN activity based on electrochemiluminescence immunoassay techniques. All the observations were measured and analyzed on the cobas 8000 platform (Roche Diagnostics, Shanghai, China).

### 4.13. Statistical Analysis

All the experiments were done in triplicate, and the measurement data are presented as the mean ± standard deviation (SD). The single factor analysis of variance (ANOVA) and a paired t-test were utilized for statistical comparisons. SPSS 20.0 (SPSS Inc., Chicago, IL, USA) were used for statistical analysis, and *p* < 0.05 was considered statistically significant.

## Abbreviations

ALP	alkaline phosphatase
ANOVA	analysis of variance
BMP	bone morphogenetic protein
BMP-2	bone morphogenetic protein 2
Ct	cycle threshold
GAPDH	glyceraldehyde-3-phosphate-dehydrogenase
GFP	green fluorescent protein
hAMSCs	human amnion mesenchymal stem cells
MSCs	mesenchymal stem cells
MTS	3-(4,5-Dimethylthiazol-2-yl)-5-(3-carboxymethoxyphenyl)-2-(4-sulfophenyl)-2*H*-tetrazolium
nHAC/PLA	nano-hydroxyapatite/collagen/poly(l-lactide)
OB	osteoblast
OC	osteoclast
OCN	osteocalcin
OD	optical density
PBS	phosphate buffered saline
rhBMP-2	recombinant human BMP-2
RT-qPCR	Reverse transcription quantitative real-time polymerase chain reaction
SD	standard deviation
SDS/PAGE	sodium dodecyl sulphate polyacrylamide gel electrophoresis
TGF-β	transforming growth factor-β
